# Patterns of occurrence and antimicrobial resistance of *Escherichia coli* and *Staphylococcus aureus* in public playgrounds

**DOI:** 10.1038/s41598-026-49426-x

**Published:** 2026-04-27

**Authors:** Rafał Łopucki, Marcin Skowronek, Anna Bilokinna, Aleksandra Niziołek, Ilona Sadok

**Affiliations:** 1 Department of Biomedicine and Environmental Research, Institute of Biological Sciences, Faculty of Medicine, Collegium Medicum, The John Paul II Catholic University of Lublin, Konstantynów 1J, Lublin, 20-708 Poland; 2 Faculty of Medicine, Biotechnology Scientific Club of the John Paul II Catholic University of Lublin, The John Paul II Catholic University of Lublin, Konstantynów 1I street, Lublin, 20-708 Poland; 3 Department of Microbiology, Institute of Soil Science and Plant Cultivation - State Research Institute, Czartoryskich 8, Pulawy, 24-100 Poland; 4 Department of Biomedical and Analytical Chemistry, Institute of Biological Sciences, Faculty of Medicine, Collegium Medicum, The John Paul II Catholic University of Lublin, Konstantynów 1J, Lublin, 20-708 Poland

**Keywords:** MALDI-TOF MS, Sandbox, Children, Antibiotic Resistance, *Escherichia coli*, *Staphylococcus aureus*, Environmental sciences, Microbiology

## Abstract

Public playgrounds are critical spaces for child development but may also serve as reservoirs and transmission pathways for potentially pathogenic and antimicrobial-resistant bacteria. Despite growing evidence linking urban environments with microbial hazards, systematic data on bacterial contamination and resistance profiles in playgrounds remain scarce. To address this gap, 33 playgrounds in Lublin, Poland, were screened for *Escherichia coli* and *Staphylococcus aureus*, including antibiotic-resistant strains. In total, 330 environmental samples were collected, and statistical analyses assessed associations between bacterial contamination and specific playground attractions or substrate materials. *E. coli* and *S. aureus* were detected in 50 (15%) and 15 (4.5%) samples, respectively. The presence of *E. coli* was strongly associated with sandboxes. For *S. aureus*, the highest risk was linked to structures touched by hand. Antibiotic resistance profiling revealed that among 80 *E. coli* isolates, resistance was highest to ampicillin (32.5%), followed by tetracycline and sulfamethoxazole (23.8%). Moderate resistance was observed for trimethoprim (20%), streptomycin (18.8%), ciprofloxacin (17.5%) and trimethoprim-sulfamethoxazole (16.2%). For *S. aureus*, resistance was highest to penicillin (86.7%), followed by erythromycin (13.3%). Multidrug resistance occurred in 21.3% of *E. coli* isolates, with no association with location or material. These findings underscore the need to monitor playground bacterial contamination and promote hygiene awareness in public spaces.

**Supplementary Information:**

The online version contains supplementary material available at 10.1038/s41598-026-49426-x.

## Introduction

The extensive use of antibiotics in human medicine, veterinary practice, and agriculture has accelerated the spread of antimicrobial resistance (AMR). This phenomenon is a predictable outcome of microbial evolution, in which genetic variation and selection under antimicrobial pressure favour bacterial populations able to survive exposure to antibacterial agents. As a result, microorganisms acquire and maintain defence mechanisms that can progressively reduce the effectiveness of therapy^1^. Antibiotic-resistant infections have therefore become a major therapeutic challenge, increasing the likelihood of treatment failure, severe complications, prolonged hospitalization, and death. The worldwide expansion of multidrug-resistant bacteria - often referred to “superbugs” - has been described as a silent pandemic and current projections indicate that by 2050 AMR may account for up to 10 million deaths annually, exceeding mortality caused by cancer and cardiovascular disease^2^.

Although much of the AMR literature has focused on hospitals and other clinical environments, resistant bacteria circulate across a much broader ecological network. Within the One Health framework, environmental settings are increasingly recognized as important interfaces linking human, animal, and abiotic reservoirs of microorganisms and resistance traits^3–5^. Urban public spaces are particularly relevant in this regard because they are repeatedly exposed to human contact, environmental deposition, animal activity, and fluctuating weather conditions, all of which may influence microbial persistence and transfer^6,7^. However, compared with healthcare institutions, such sites remain less frequently monitored from a microbiological perspective^3,6^.

Among urban public environments, playgrounds warrant particular attention. They are intensively used by children, a population characterized by frequent hand-to-mouth behaviour, close contact with shared surfaces, and repeated exposure to sand, soil, and dust. At the same time, playground equipment and loose substrate materials are touched by many users every day, often with only limited routine hygienic control. These settings may therefore function as environmental interfaces where microorganisms originating from human skin, respiratory secretions, soil particles, dust, animal faeces, and other urban sources accumulate on high-contact surfaces and loose-fill materials^8–10^. Sandboxes, swings, climbing structures, benches, spring riders, and ground coverings may thus serve as transient reservoirs of bacteria introduced directly by users or indirectly through companion animals, wild birds, and environmental deposition. Such environmental relevance is consistent with previous reports demonstrating the recovery of *Staphylococcus aureus*, including methicillin-resistant *Staphylococcus aureus* (MRSA), from children’s playground surfaces in different regions^11,12^.

This rationale is further supported by previous observations indicating that playground sandboxes and adjacent equipment may become contaminated with faecal material from dogs, cats, and free-living birds^13–15^. Dog faeces have been identified as a potentially important reservoir of antibiotic-resistant *Escherichia coli* in public environments^16–18^ while companion animals may also carry and shed *S. aureus*, including MRSA strains, creating opportunities for contamination of frequently touched objects and loose substrates^19,20^. Wild birds visiting urban recreational areas may likewise disseminate resistant *E. coli* through faecal deposition^21–23^. Because children’s hands often serve as intermediaries in the transfer of enteric and opportunistic bacteria^24,25^, playgrounds represent a plausible point of environmental exposure that deserves focused microbiological investigation^10^.

Once introduced, bacteria may persist on playground materials for hours to weeks, particularly on plastics and metals that can support adhesion and biofilm formation^26^. In addition, children may preferentially interact with plastic and wooden equipment, potentially increasing contact frequency with these surfaces^27^.

In this context, *E. coli* and *S. aureus* are suitable indicator organisms for baseline environmental screening. *E. coli* is widely used as a marker of faecal contamination and may reflect the introduction of enteric bacteria into playground environments via animal faeces, contaminated soil, or direct human activity. Importantly, some environmental *E. coli* strains may also harbour clinically relevant resistance phenotypes, making this species informative in studies addressing the spread of AMR beyond healthcare settings. Clinically important strains, including ESBL-producing and carbapenem-resistant *E. coli* isolates, are of particular concern because they are associated with limited therapeutic options and increased public-health relevance^28,29^.

By contrast, *S. aureus* is primarily associated with human and animal skin and mucosal colonization and is readily transmitted by direct contact. Its detection on playground equipment may therefore indicate contamination of high-touch surfaces repeatedly handled by children and caregivers. From a clinical perspective, *S. aureus* remains one of the most important human pathogens, particularly in the context of methicillin resistance^30–33^. Considered together, these two bacterial species provide complementary insight into two epidemiologically relevant contamination routes in public play areas: faecal-environmental introduction and contact-associated surface contamination.

Despite growing awareness of environmental AMR, systematic data on the occurrence of *E. coli* and *S. aureus* in playgrounds remain limited and methodologically heterogeneous^10^. Available studies differ in sampling strategy, the categories of surfaces examined, the range of microorganisms targeted, and the extent to which resistance phenotypes are characterized. As a result, comparative information remains scarce regarding how frequently these organisms occur across different types of playground equipment and substrate materials, particularly in urban settings of Central and Eastern Europe^10^. This gap constrains the evidence base needed to interpret playgrounds as components of the wider urban AMR landscape and to formulate rational recommendations for environmental hygiene and risk reduction.

Therefore, the aim of this study was to perform a baseline environmental screening of public playgrounds in Lublin, Poland, in order to assess the occurrence of *E. coli* and *S. aureus* on selected playground equipment and substrate materials and to characterize the antimicrobial susceptibility profiles of the recovered isolates. We hypothesized that the frequency of bacterial detection would vary between sampling locations and material categories, with higher detection expected in loose substrates and on frequently touched surfaces, and that at least a proportion of environmental isolates would exhibit phenotypic resistance to one or more antimicrobial agents. Because the study was observational and descriptive, it was designed to identify occurrence patterns and associations rather than to infer causal determinants.

## Methods and Materials

### Playground Selection and Sampling Protocol

**Fig. 1:**
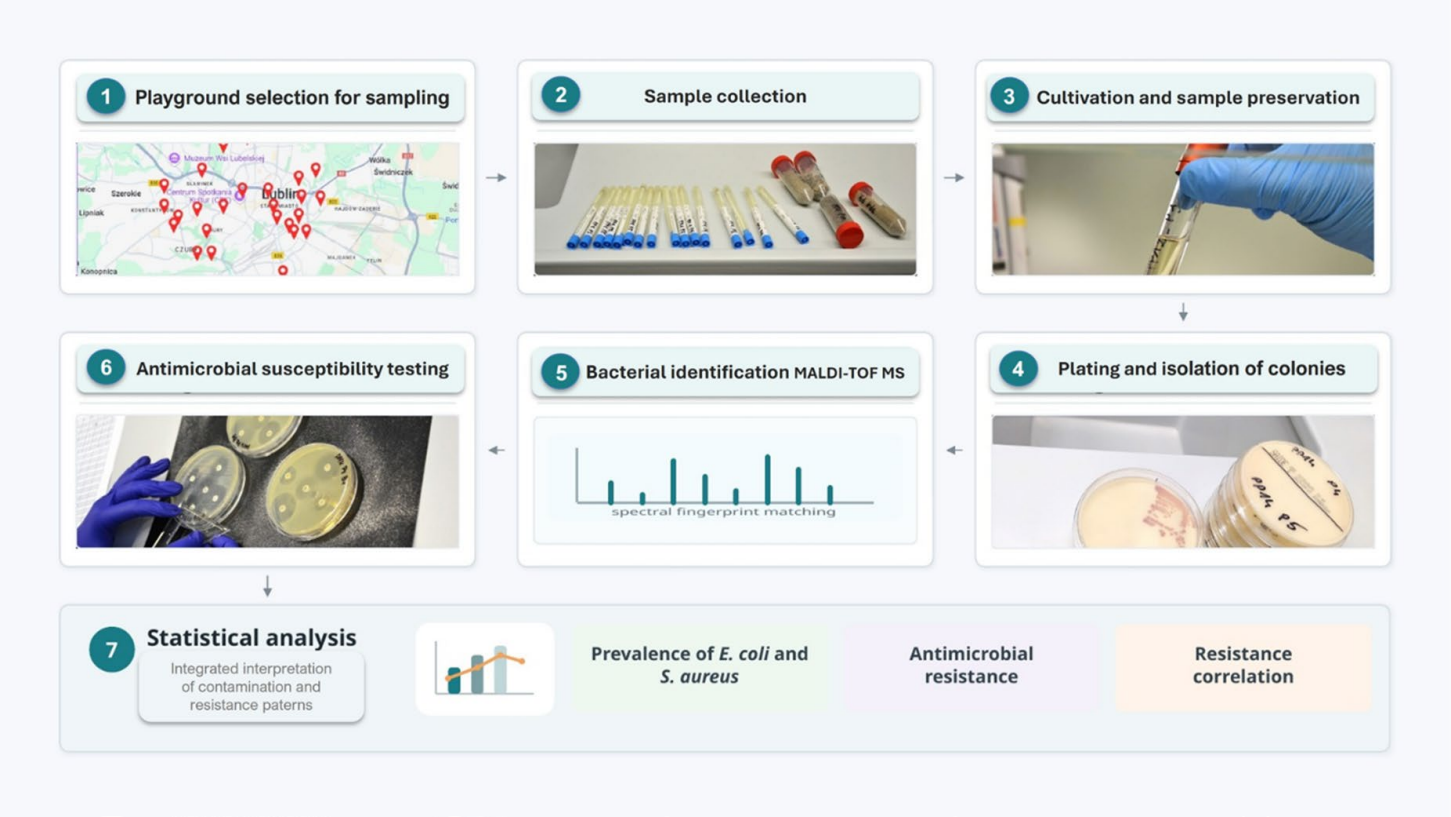
Flowchart presenting the study workflow.

The workflow applied in this study is presented in Fig. 1. All playgrounds included in the study were located within the city of Lublin, Poland. Selection for microbiological monitoring was based on user popularity, public accessibility, and diversity of equipment. Playgrounds situated within kindergartens or on private, gated estates were excluded. Locations were further chosen to ensure even spatial distribution across the city’s districts. The locations of playgrounds are shown in Supplementary Figure S1. The geographical coordinates are provided in the corresponding dataset available in the OSF repository (https://osf.io/8jsk4/). Ultimately, 33 playgrounds were included in the study.

Samples were collected from 10 different locations at each playground, including both equipment elements and the dominant type of surface for the respective site. In total, 330 environmental samples were obtained, which were subsequently subjected to further laboratory analysis. General types of sampling locations included: swing, climbing structures, bench, ground surface, spring rider/other, and sandbox. General types of materials included: plastic, metal and mixed materials, natural materials, sand, soil, and rubber safety surfaces. A detailed list of samples collected from each playground, including the type of equipment and the dominant substrate material, is provided in the dataset available in the OSF repository (https://osf.io/8jsk4/).

Sample collection was carried out in June and July 2023 during evening hours, following periods of intensive playground use by children, in accordance with the previously established methodology^34^. Weather conditions were considered when scheduling sampling to ensure accuracy; samples were collected only on dry days to prevent rainfall from affecting bacterial counts on surfaces.

Surface samples from playground equipment and ground coverings were collected using sterile swabs moistened with sterile saline solution (0.9%). Targeted surfaces were swabbed for two minutes, after which swabs were placed in transport tubes and stored in a refrigerated container at approximately 4 °C prior to immediate laboratory transport.

For soil, sand, and gravel samples, material was collected into sterile 50 mL containers. Each composite sample was obtained from three distinct points within the same playground to ensure representativeness. All procedures were performed using protective gloves and sterile equipment to minimize the risk of sample contamination.

### Bacterial Sample Processing and Isolation Procedures

All microbiological manipulations were performed under a laminar flow cabinet. Cotton swab tips were aseptically cut and immersed in 5 mL of sterile tryptic soy broth (TSB, BTL, Poland). Soil and sand samples were suspended in 100 mL of sterile TSB. All cultures were incubated overnight at 37 °C to allow bacterial pre-enrichment. Following pre-enrichment, bacterial suspensions were streaked with sterile inoculation loops onto selective chromogenic agar media. For the isolation of *E. coli*, Chromogenic Coliform Agar (CCA; BTL, Poland) and chromID CPS Elite agar (bioMérieux, France) were used, whereas *S. aureus* was isolated on chromID *S. aureus* Elite agar (SAIDE; bioMérieux, France). All plates were incubated at 37 °C for 24–48 h under aerobic conditions. Representative colonies displaying characteristic morphologies and colours were selected according to the manufacturer’s guidelines for each medium. Single colonies were transferred into 5 mL of sterile TSB and incubated overnight at 37 °C. Pure cultures were either processed for further identification or supplemented with 20% (*v/v*) glycerol and stored at -80 °C for subsequent use.

### Species-level Identification of Bacteria by MALDI-TOF MS

Bacterial isolates were identified using matrix-assisted laser desorption/ionization time-of-flight mass spectrometry (MALDI-TOF MS). For analysis, a single colony was suspended in 100 µL of sterile ultrapure water, followed by 300 µL of ethanol (Witko, Poland), vortexed, and centrifuged at 13,000 rpm for 5 min (Eppendorf 5415R, Germany). The supernatant was discarded, and the pellet was dried at 30 °C using an EZ-2 Elite Personal Evaporator (Genevac Ltd, UK). The pellet was then resuspended in 40 µL of 70% (*v/v*) aqueous formic acid (Merck, Germany), vortexed for 3 min, and mixed with the same volume of acetonitrile (Merck, Germany), followed by centrifugation for 3 min at 13,000 rpm.

An aliquot of 1 µL of the extract was spotted in triplicate onto a polished steel MALDI target plate, overlaid with 1 µL of saturated α-cyano-4-hydroxycinnamic acid (HCCA, Bruker Daltonik GmbH, Germany) in matrix solution (50% acetonitrile, 47.5% ultrapure water, 2.5% trifluoroacetic acid), and air-dried.

Samples loaded onto a MALDI plate were analyzed using an UltrafleXtreme MALDI-TOF MS system (Bruker Daltonik GmbH, Germany). Spectra were acquired in linear positive-ion mode across an m/z range of 2,000–20,000 Da, with laser energy set at 65–75% and a minimum of 1,500 laser shots applied. Mass calibration was performed using the Bacterial Test Standard. Spectra were analyzed with MALDI Biotyper Compass Explorer 4.1. Identification scores were interpreted as follows: <1.70, unreliable; 1.70–1.99, probable genus; 2.00-2.29, secure genus/probable species; 2.30-3.00, highly probable species. A result was considered reliable if the two highest-scoring matches corresponded to the same species; in cases of discrepancy, the species with the higher log(score) was selected.

### Antibiotic Susceptibility Testing

The antibiotic susceptibility of the isolated bacterial strains was evaluated using the Kirby-Bauer disk diffusion method on Mueller-Hinton agar (MHA; Merck, Germany). Bacterial inocula were prepared from 18 to 24 h colonies suspended in sterile saline to a turbidity equivalent to the 0.5 McFarland, and the inoculated plates were incubated at 35 °C for 16–18 h. Disk diffusion testing was performed according to recommendations of the Clinical and Laboratory Standards Institute (CLSI)^35^, and inhibition zone diameters were finally interpreted according to CLSI^36^. All assays were performed in triplicate.

In total, 14 antibiotics were tested against *E. coli* isolates (Table 1) and 11 against *S. aureus* isolates (Table 2). The *E. coli* panel was selected to provide broad phenotypic coverage of the major antimicrobial classes commonly used for resistance screening in enteric Gram-negative bacteria, including β-lactams, aminoglycosides, tetracycline, sulfonamides/trimethoprim, and fluoroquinolones. Additionally, a cefotaxime disk supplemented with clavulanic acid (CTL, 40 µg) was used to assess extended-spectrum β-lactamase (ESBL) production by comparing the inhibition zone diameter with that of a cefotaxime-only disk (difference ≥ 5 mm)^36^. The *S. aureus* panel was selected to capture key phenotypic resistance patterns in staphylococci by including agents from major antimicrobial classes relevant to Gram-positive bacteria, together with selected compounds informative for staphylococcal susceptibility profiling. After incubation, the diameters of the inhibition zones were measured to the nearest millimetre. Because the analyzed isolates were of environmental origin, clinical breakpoint-based classification was used to enable standardized phenotypic comparison of susceptibility profiles, while acknowledging the limitations of applying clinical interpretive criteria to non-clinical strains.

The following reference strains were used for validation: *E. coli* ATCC 25,922 (susceptible quality-control strain), *E. coli* NCTC 13,353 (CTX-M-15 ESBL-producing strain), *E. coli* NCTC 13,476 (β-lactam-resistant reference strain), *S. aureus* ATCC 25,923 (susceptible quality-control strain), and *S. aureus* ATCC 700,699 (MRSA reference strain). All strains were purchased from Microbiologics (Glasgow, UK).

### Statistical Analyses

Statistical analyses were performed to assess whether bacterial occurrence, antimicrobial resistance, and multidrug resistance varied according to playground sampling location and material type. Because several outcomes were sparse, particularly for *S. aureus* and for some resistance phenotypes, Bayesian generalized linear mixed-effects models (GLMMs) with a logit link were used to obtain stable estimates and probabilistically interpretable uncertainty intervals. The general modelling framework, including detailed model equations, prior specifications, MCMC settings, and convergence diagnostics, is provided in the Supplementary Methods S1-S6.

#### Occurrence Models

For bacterial occurrence, separate Bayesian GLMMs were fitted for *E. coli* and *S. aureus*, with detection status (present/absent) as the binary response. Fixed effects included sampling location and material type, and a random intercept for playground was included to account for clustering of samples within playgrounds. Interactions between sampling location and material type were not analysed because several cross-classified categories were sparse. Results are reported as odds ratios (OR) with 95% credible intervals (CrI). Detailed model specification is provided in Supplementary Methods S2.

#### Antimicrobial Resistance and Multidrug Resistance Models

Antimicrobial susceptibility data were first summarized descriptively as the numbers of isolates classified as susceptible, intermediate, or resistant for each antimicrobial agent. Because only a small number of *S. aureus* isolates were recovered, inferential resistance analyses were restricted to *E. coli*. For antimicrobial agents with at least 10 resistant isolates, resistance was modelled as a binary outcome (R vs. S/I) using separate Bayesian GLMMs with sampling location and material type as fixed effects. Random intercepts for playground and sample were included because multiple *E. coli* isolates were sometimes recovered from the same environmental sample. Detailed resistance model specification is provided in Supplementary Methods S3.

Multidrug resistance (MDR) analysis was also restricted to *E. coli*. Isolates were classified as MDR if they were resistant to at least one agent in three or more antimicrobial classes. The probability of MDR was then analysed using a Bayesian GLMM with the same fixed effects and random-effects structure as in the resistance models. Results are presented as OR with 95% CrI. Detailed MDR model specification is provided in Supplementary Methods S4.

#### Resistance Correlation Analysis

Patterns of co-resistance among *E. coli* isolates were explored using binary resistance data (R vs. S/I). Antimicrobial agents with ≤ 3 resistant isolates were excluded to reduce instability caused by sparse categories. Pairwise phi correlations between resistance indicators were calculated and converted into a dissimilarity matrix for hierarchical clustering. The resulting clusters were visualized in a correlation heatmap. In parallel, multiple correspondence analysis (MCA) was performed on the same binary resistance matrix to visualize relationships among resistance phenotypes in a low-dimensional space. Additional details are provided in Supplementary Methods S5.

#### Model fitting and reporting

Models were fitted in R using the brms package interfaced with Stan, with weakly informative priors applied throughout. Convergence and sampling efficiency were assessed using standard MCMC diagnostic criteria. Results are presented as OR with 95% CrI and visualized using forest plots where appropriate. Additional details on priors, model fitting, diagnostics, and software are provided in Supplementary Methods S6.

## Results

### Presence of *E. coli* in Playground Samples

**Fig. 2:**
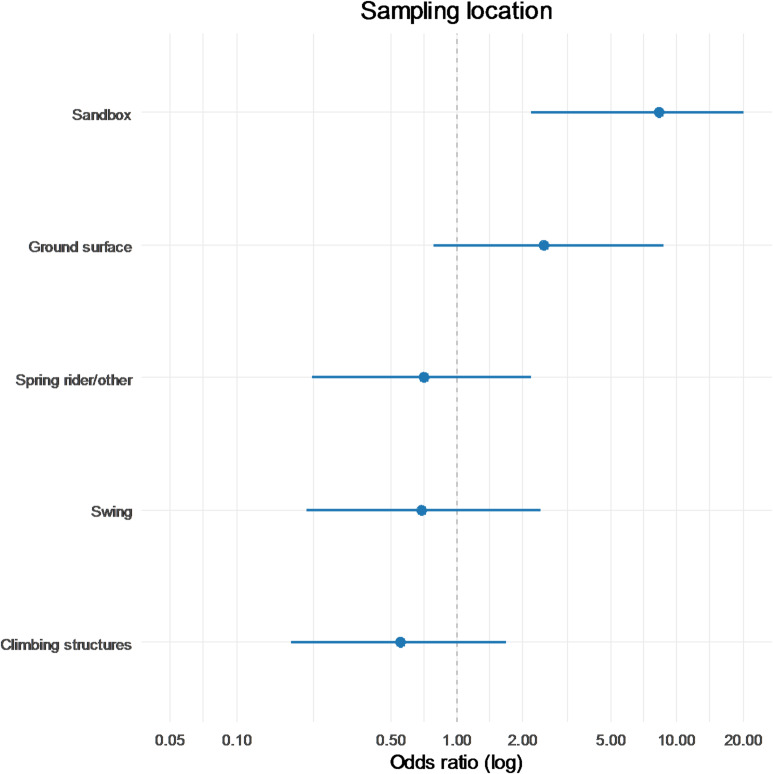
Posterior odds ratios (ORs) for *E. coli* detection by sampling location, estimated using a Bayesian generalized linear mixed model (GLMM). Points represent posterior means; horizontal bars indicate 95% credible intervals (CrI). The vertical dashed line at OR = 1 marks the null effect. Sandboxes were associated with a markedly higher probability of *E. coli* presence, while other locations showed weaker (Ground surface) or non-significant associations (climbing structures, swings, and spring riders/other attractions).

Out of all 330 collected samples, *E. coli* was recovered from 50 cases, representing 15% of the total. The GLMM analysis showed that the location of the samples had a significant effect on *E. coli* prevalence (Fig. 2). Compared with the reference level (Bench), samples taken from sandboxes were eight times more likely to yield *E. coli* (OR = 8.3; 95% CrI = 2.1–32). Ground surface samples also showed a positive but weaker effect (OR = 2.5; CrI = 0.74–8.3), while samples from climbing structures, swings, and spring riders/other attractions were comparable to the reference (CrI widely overlapping 1).

**Fig. 3:**
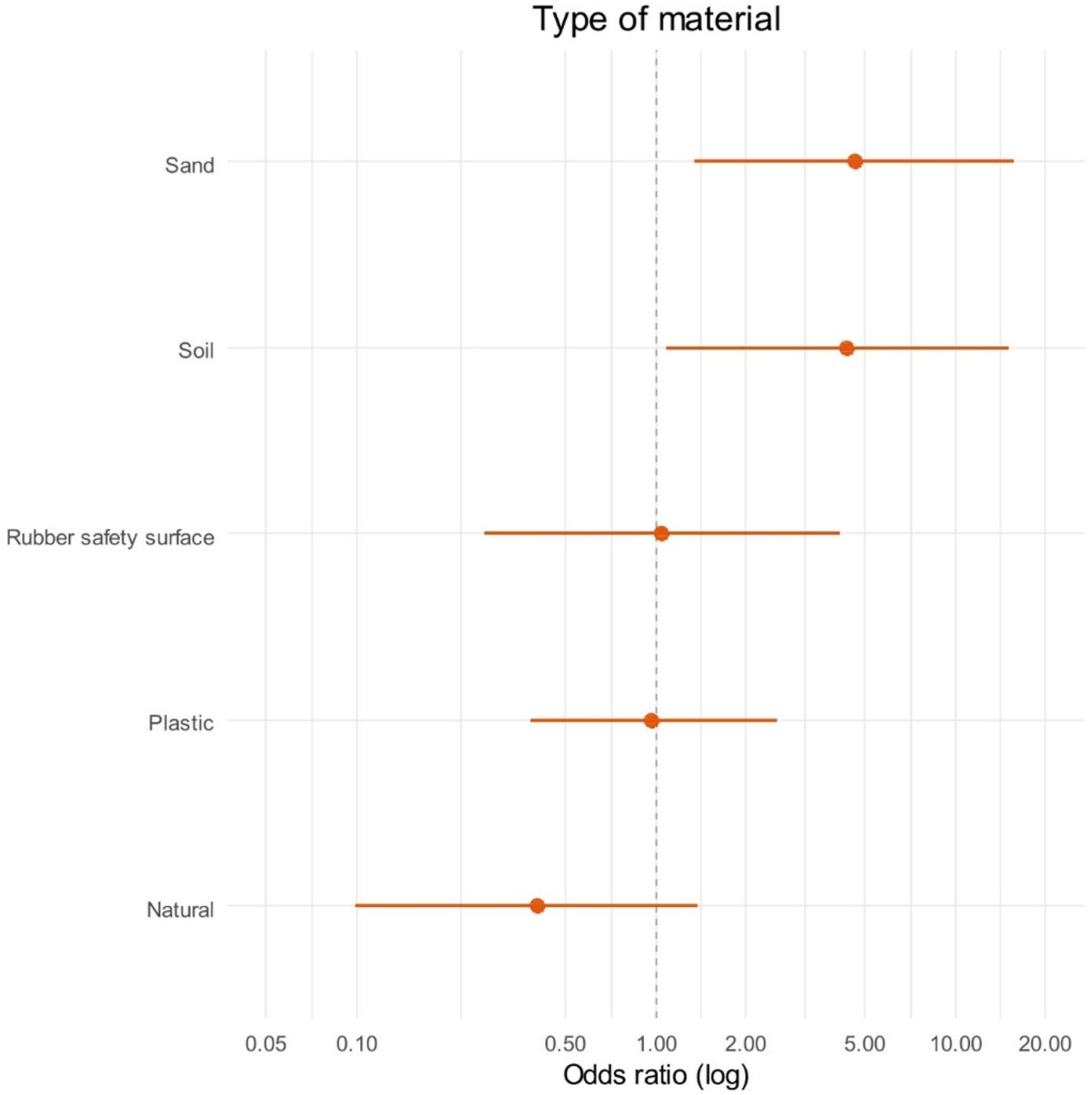
Posterior odds ratio (OR) estimates from a Bayesian generalized linear mixed model (GLMM) for *E. coli* detection by substrate material, relative to the reference category (metal and mixed surfaces - not shown on the plot). Points indicate posterior means, and horizontal lines represent 95% credible intervals (CrI); the dashed line at OR = 1 denotes no effect. Sand and soil were significantly associated with increased likelihood of *E. coli* presence, while natural materials (e.g., untreated wood, ropes) showed a potential protective effect (but the upper CrI limit approaching unity weaken this conclusion). Plastic and rubber surfaces did not differ significantly from the reference.

Substrate material also had an effect (Fig. 3). Relative to metal and mixed surfaces (reference), soil (OR = 4.3; CrI = 1.1–16) and sand (OR = 4.6; CrI = 1.4–15.8) markedly increased the probability of *E. coli* isolation. Natural materials (unpainted wood or natural ropes) tended to be protective (OR = 0.40), although the small sample size and the upper CrI limit approaching unity (0.10–1.44) limit the strength of this conclusion. Plastic and rubber safety surfaces did not differ appreciably from the reference (OR ≈ 1; CrI wide and crossing 1).

Convergence of all fixed-effect parameters in the Markov chain Monte Carlo (MCMC) analysis was satisfactory, with R̂ values ≤ 1.01 and bulk effective sample sizes (ESS) exceeding 1,000. The estimated standard deviation of the random intercept for playground ID (posterior mean = 1.1) suggests substantial between-playground heterogeneity not fully accounted for by the fixed effects (sampling location and material type). This indicates the presence of unmeasured playground-level factors influencing contamination outcomes. Posterior predictive performance was satisfactory, with an area under the receiver operating characteristic curve (AUC) of 0.84, indicating good discriminative ability of the model in predicting the presence or absence of *E. coli* across different playground features.

### Presence of *S. aureus* in Playground Samples

Out of the 330 samples collected, *S. aureus* was detected in 4.5% of them, i.e. in 15 samples. The lLow number of positive samples meant that none of the tested variables showed a statistically credible association with *S. aureus* presence: all CrIs included the null value (OR = 1), suggesting that the model lacked the power to identify significant predictors.

**Fig. 4:**
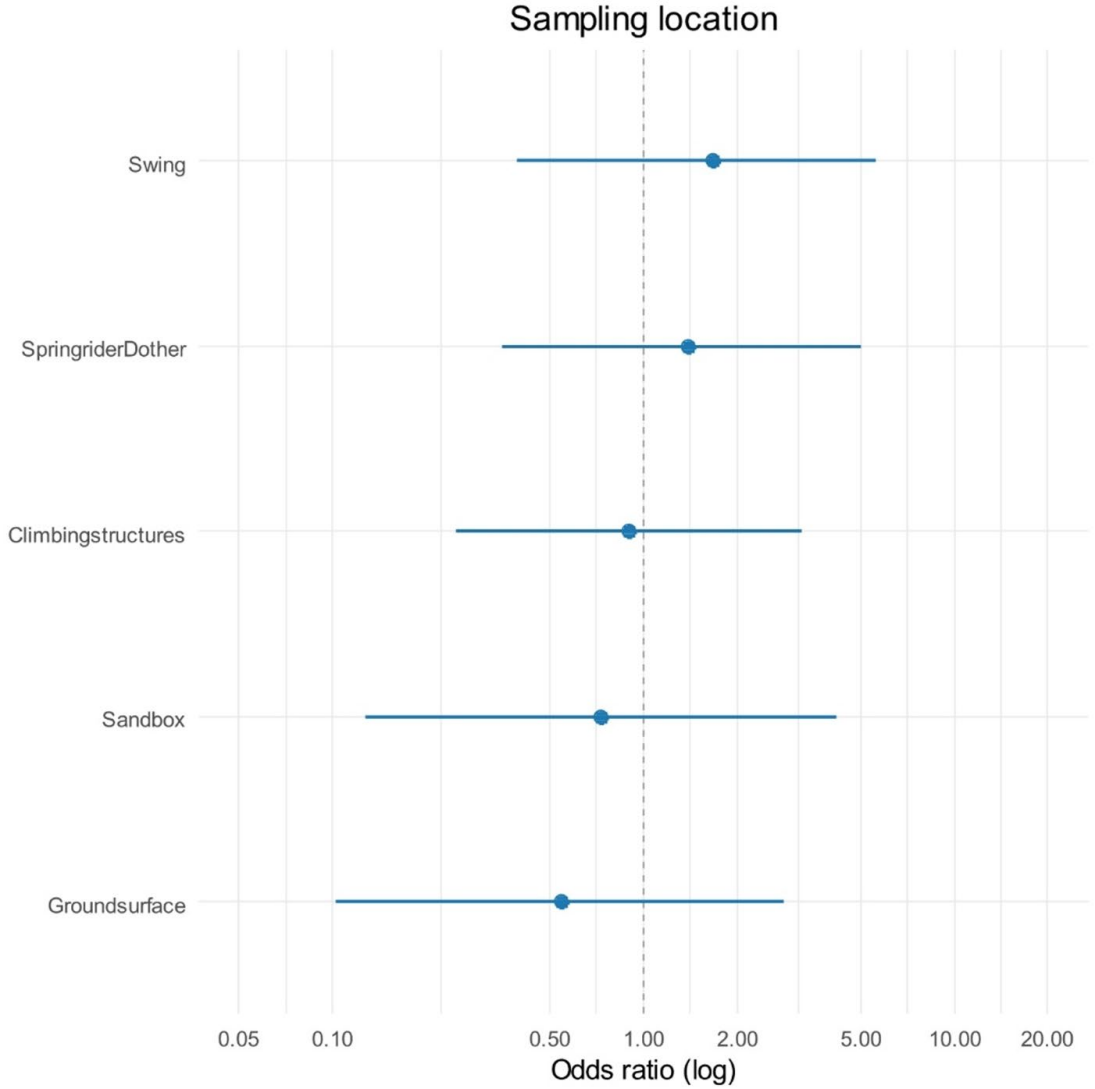
Posterior odds ratios (ORs) for *S. aureus* detection by sampling location, estimated using a Bayesian GLMM. Points represent posterior means; horizontal lines indicate 95% credible intervals (CrI). Swing structures and spring riders had the highest estimated ORs, but none of the locations showed statistically credible associations, as all intervals overlapped 1.

Among sampling locations (Fig. 4), the highest point estimates of risk were observed for swing structures (OR = 1.67; CrI = 0.44–6.26) and spring riders or other attractions (OR = 1.39; CrI = 0.35–5.14), which are frequently touched by hand. However, the wide CrIs indicate considerable uncertainty. Locations such as climbing structures, sandboxes, and ground surfaces yielded ORs below 1, but again with non-informative intervals.

**Fig. 5:**
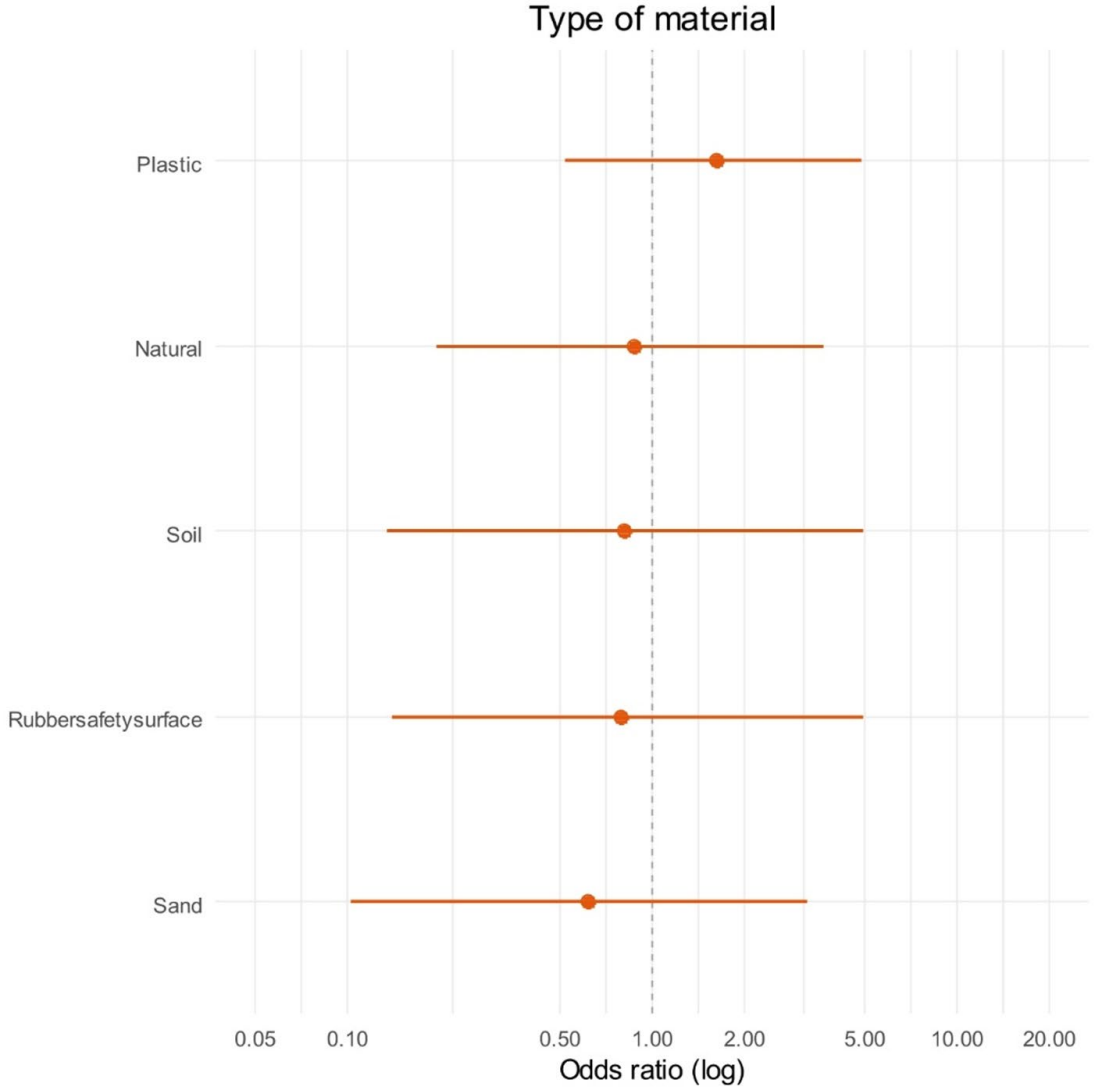
Posterior odds ratios (ORs) for *S. aureus* detection by surface material, estimated using a Bayesian generalized linear mixed model (GLMM). Points represent posterior means; horizontal lines denote 95% credible intervals (CrI). The dashed line at OR = 1 indicates no effect. Plastic surfaces showed the highest point estimate, but all CrIs included 1, indicating no statistically credible association with *S. aureus* presence.

In terms of surface materials (Fig. 5), none showed a consistent effect. Plastic surfaces yielded the highest OR (1.62; CrI = 0.53–5.06), while all other materials (rubber, sand, soil, natural) were associated with ORs < 1, but with wide CrIs crossing unity.

The model intercept (OR = 0.017; CrI = 0.004–0.065) reflects the very low overall prevalence of *S. aureus* in the baseline sample type (bench with metal or mixed surface).

### Antimicrobial Resistance of *E. coli*

Out of 80 *E. coli* isolates, antimicrobial resistance patterns varied notably between substances (Table 1). Ampicillin (AMP10) showed the highest resistance (32.5%), followed by sulfamethoxazole (S3-300) and tetracycline (TE30), both at 23.8%. Moderate resistance was observed for trimethoprim (W5, 20%), streptomycin (S10, 18.8%), ciprofloxacin (CIP5, 17.5%) and trimethoprim-sulfamethoxazole (SXT25, 16.2%). In contrast, resistance to amoxicillin-clavulanate (AMC30), cefotaxime (CTX30), chloramphenicol (C30), kanamycin (K30), gentamicin (CN10), and carbapenems (MEM10, IPM10) was rare or absent. ESBL-producing isolates accounted for 6.25% of *E. coli* isolates (5 out of 80).

**Table 1:** Number and percentage of *E. coli* isolates (*n* = 80) classified as resistant to each tested antimicrobial agent. Agents are listed in descending order of resistance prevalence. Only antimicrobial agents with ≥ 10 resistant isolates were included in subsequent statistical modelling.

Antimicrobial agent	No. of resistant isolates	% of resistant isolates (*n* = 80)
AMP10	26	32.5%
S3-300	19	23.8%
TE30	19	23.8%
W5	16	20.0%
S10	15	18.8%
CIP5	14	17.5%
SXT25	13	16.2%
AMC30	4	5.0%
C30	4	5.0%
CTX30	4	5.0%
K30	4	5.0%
CN10	1	1.2%
MEM10	1	1.2%
IPM10	0	0%

AMC30 - amoxicillin-clavulanic acid 30 µg; AMP10 - ampicillin 10 µg; C30 - chloramphenicol 30 µg; CIP5 - ciprofloxacin 5 µg; CN10 - gentamicin 10 µg; CTX30 - cefotaxime 30 µg; IPM10 - imipenem 10 µg; K30 - kanamycin 30 µg; MEM10 - meropenem 10 µg; S10 - streptomycin 10 µg; S3-300 - sulfamethoxazole 300 µg; SXT25 - trimethoprim-sulfamethoxazole 25 µg; TE30 - tetracycline 30 µg; W5 - trimethoprim 5 µg;

To investigate whether resistance patterns in *E. coli* were associated with sampling location or substrate material, Bayesian generalized linear mixed models (GLMMs) were fitted separately for antimicrobial agents with at least 10 resistant isolates (see Table 1).

None of the modelled antimicrobial agents showed statistically credible associations between resistance and tested variables. All 95% credible intervals (CrI) for odds ratios included the null value (OR = 1), indicating that observed differences may reflect random variation rather than systematic effects. As illustrative examples, we present full model results for AMP10 (Supplementary Figure S2) and CIP5 (Supplementary Figure S3), two agents with relatively high resistance prevalence (33% and 17.5%, respectively). These models reflect the general pattern observed across all substances.

### Antimicrobial Resistance of *S. aureus*

The resistance profiles of the 15 environmental *S. aureus* isolates are summarized in Table 2. The highest resistance rate was observed for penicillin, with 13 out of 15 isolates (86.7%) classified as resistant. Two isolates (13.3%) were resistant to erythromycin, while all other tested agents showed full activity, with 100% of isolates classified as susceptible. Due to the low number of *S. aureus* isolates recovered, no statistical modelling of resistance determinants was performed for this bacterium.

**Table 2:** Resistance of *S. aureus* isolates (*n* = 15) to tested antimicrobial agents. The table shows the number and percentage of resistant isolates per antimicrobial agent.

Antimicrobial agent	No. of resistant isolates	% of resistant isolates (*n* = 15)
P10	13	86.7%
E15	2	13.3%
OB5	0	0%
KZ30	0	0%
CPT5	0	0%
DA2	0	0%
CIP5	0	0%
TE30	0	0%
RD5	0	0%
CN10	0	0%
LZD30	0	0%

CIP5 - ciprofloxacin 5 µg; CN10 - gentamicin 10 µg; CPT5 - ceftaroline 5 µg; DA2 – clindamycin 2 µg; E15 - erythromycin 15 µg; KZ30 - cefazolin 30 µg; LZD30 - linezolid 30 µg; OB5 - cloxacillin 5 µg; P10 - penicillin 10 µg; RD5 - rifampicin 5 µg; TE30 – tetracycline 30 µg.

### Multidrug Resistance of *E. coli*

Among the 80 *E. coli* isolates, 17 (21.3%) met the MDR definition (resistant to ≥ 3 antimicrobial classes). Mixed model estimates showed that none of the sampling locations or material types showed a statistically credible association with MDR: for all fixed effect contrasts the 95% CrI for the odds ratio included 1 (Supplementary Figure S4). The largest, yet non-decisive, point estimate was observed for sand samples (OR ≈ 2.1; 95% CrI 0.6–7.4). The posterior standard deviation for the sample level random effect was 1.05 (95% CrI 0.62–1.73), indicating considerable heterogeneity between samples, whereas the playground level variability was modest (sd = 0.42; 95% CrI 0.15–0.98). The substantial residual variability at the sample level suggests that MDR occurrence may be driven by sporadic, highly contaminated spots rather than by consistent characteristics of particular playgrounds.

### Resistance Correlation Analysis

**Fig. 6:**
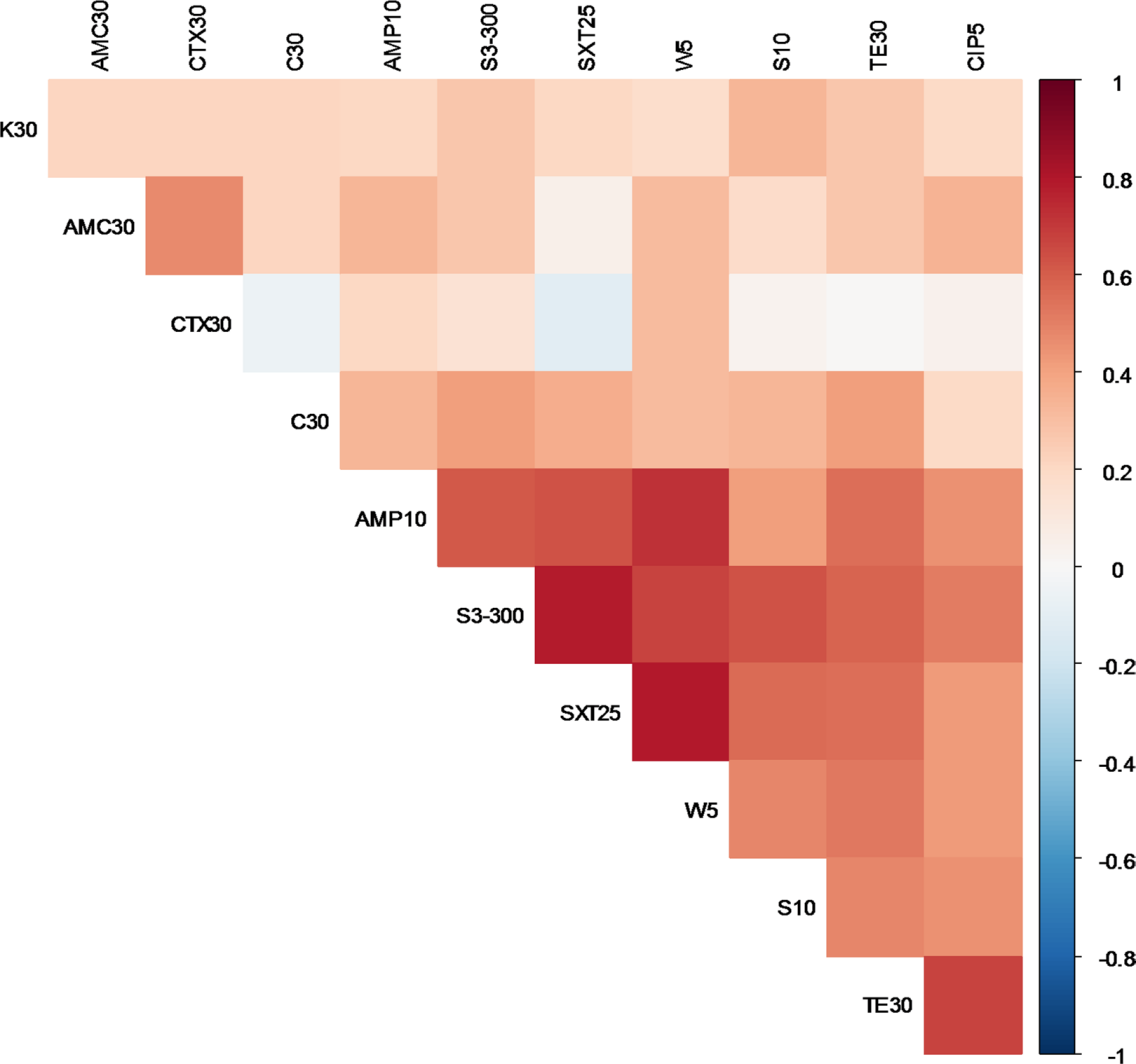
Pairwise correlation heatmap of antibiotic resistance phenotypes. Strong positive correlations (in red) indicate co-resistance patterns, while white and blue tones represent weak or negative associations.

Both the correlation heatmap (Fig. 6) and the MCA biplot (Supplementary Figure S5) consistently revealed three clear clusters of co-resistance. The first, representing a classic multidrug-resistant (MDR) pattern, included ampicillin, tetracycline, various trimethoprim-sulfonamide combinations (S3-300, SXT25, W5), streptomycin, and ciprofloxacin. These antimicrobial agents showed strong mutual correlations (φ ≥ 0.60) and grouped closely together along the higher end of Dimension 1 in the MCA (Supplementary Figure S5). The second cluster reflected associations among β-lactam-related drugs: AMC30, K30, CTX30. They demonstrated moderate correlations (φ ≈ 0.3–0.5) and were grouped as a distinct cluster in the upper-right quadrant of the MCA plot. The third and final cluster consisted solely of chloramphenicol, which behaved independently from all other antimicrobial agents (φ < 0.3) and was clearly separated from the other groups in both visualizations.

## Discussion

In this study, we attempted to evaluate the prevalence and occurrence patterns of *E. coli* and *S. aureus* contamination across different playground components. *E. coli* was detected in 15% of samples collected from various playground components, with the highest prevalence observed in sandbox sand and soil from play areas. Previous research reported a lower prevalence of *E. coli* in playgrounds in Ankara, Turkey (7%), with similar frequencies in soil and swab samples^37^. Furthermore, in our study, the prevalence of *S. aureus* (4.5%) was comparable to that reported in Hungary (2.81%)^11^, but markedly lower than in a study from Ohio (USA), where it reached 31.8%^12^.

Beyond assessing the prevalence of *E. coli* and *S. aureus*, evaluating their antibiotic susceptibility is important because it provides insight into the environmental occurrence and circulation of antimicrobial-resistant isolates recovered from playground settings. A limitation of this study is that antimicrobial susceptibility results were interpreted using clinical breakpoints, although the analyzed isolates were of environmental origin; therefore, these categories should be understood primarily as standardized phenotypic classifications rather than direct predictors of clinical treatment outcome. In this study, 86.7% of *S. aureus* isolates were resistant to penicillin (Table 2). These findings are consistent with national data, which indicate that 80–90% of all *S. aureus* isolates exhibit resistance to natural penicillins, typically due to the presence of the *blaZ* gene encoding β-lactamase. In this context, penicillin resistance among environmental strains appears to be common and predictable^38^. A similar proportion of penicillin-resistant *S. aureus* isolates was reported in Hungary^11^, whereas in Ohio, all strains were penicillin-resistant^12^.

Our study also revealed that 13.3% of *S. aureus* isolates were resistant to erythromycin, while all isolates were susceptible to clindamycin (Table 2). Similar findings have been reported for Hungarian playgrounds^11^, although that study also detected inducible resistance to clindamycin. In contrast, in Ohio, 26.8% of *S. aureus* isolates were resistant to erythromycin, and 19.5% to clindamycin^12^. Furthermore, similar to the Hungarian study^11^, we did not observe phenotypic evidence suggestive of methicillin resistance or multidrug resistance among the recovered *S. aureus* isolates within the antimicrobial panel applied in this study. However, because cefoxitin or oxacillin screening disks were not included and *mecA*/*mecC* genes were not tested, the absence of MRSA cannot be concluded definitively and should be interpreted with caution. This observation is nevertheless noteworthy in the Polish context, as according to the National Medicines Institute, the prevalence of MRSA infections in Poland remained at 13.3% in 2022^38^. In contrast to findings from the United States, all isolates in this study were susceptible to tetracycline, rifampicin, gentamicin, ciprofloxacin, and linezolid. All analyzed strains were also susceptible to ceftaroline. Nevertheless, susceptibility to this agent does not by itself establish methicillin susceptibility and therefore cannot serve as a definitive basis for excluding MRSA. Although these results are encouraging, it should be noted that cases of glycopeptide-resistant strains (VISA, VRSA) and linezolid-resistant *S. aureus* (LRSA) have already been reported in Poland^38^. These “alarm pathogens” significantly limit therapeutic options.

In the present study, *E. coli* isolates from public playgrounds exhibited resistance at varying frequencies to several commonly used antimicrobial agents (Table 1). The observed resistance profile is consistent with those typically reported for environmental *E. coli* isolates and is in agreement with findings from other studies^37,39^. The highest resistance rate among *E. coli* isolates was observed for ampicillin (32.5%), whereas resistance to other β-lactam antibiotics was less frequent (≤ 5%). With regard to carbapenems, we detected a low resistance rate to meropenem (1.2%), while all isolates obtained from public playgrounds remained susceptible to imipenem. The *E. coli* strains also exhibited high resistance rates to antibiotics from the sulfonamides/trimethoprim group, including sulfamethoxazole (23.8%), trimethoprim (20%), and trimethoprim-sulfamethoxazole (16.2%). Resistance to tetracycline (23.8%) was among the highest observed for *E. coli* isolates from playgrounds. Resistance to ciprofloxacin (a fluoroquinolone) was detected in 17.5% of environmental isolates. Among aminoglycosides, the highest resistance rate was recorded for streptomycin (18.8%), followed by kanamycin (5%) and gentamicin (1.2%). Finally, resistance to chloramphenicol (a representative of the phenicols group) was observed in 5% of *E. coli* isolates. The observed resistance profile of the environmental *E. coli* isolates may reflect several well-recognized mechanisms of antimicrobial resistance. In particular, the relatively high frequency of ampicillin resistance may be consistent with the production of acquired β-lactamases, whereas resistance to tetracycline may reflect the presence of tet-associated determinants. Likewise, reduced susceptibility to sulfamethoxazole/trimethoprim may be associated with resistance determinants affecting the folate pathway, while ciprofloxacin resistance may result from chromosomal mutations in quinolone target genes and/or plasmid-mediated mechanisms. Similar environmental *E. coli* isolates recovered from playground sandboxes have also been subjected to molecular characterization, supporting the relevance of such interpretations in this setting^29,39^. Studies from Graz (Austria) and Ankara (Turkey) reported generally lower resistance rates among *E. coli* isolates for several antibiotics, including ampicillin, tetracycline, and cefotaxime, with comparable or slightly variable resistance to trimethoprim-sulfamethoxazole and other agents. Notably, no resistance to carbapenems was observed in either study, with all isolates remaining susceptible to imipenem and meropenem^37,39^.

In addition, the observed co-resistance clusters suggest distinct underlying resistance mechanisms. The first MDR cluster, including ampicillin, tetracycline, trimethoprim-sulfonamide combinations (S3-300, SXT25, W5), streptomycin, and ciprofloxacin, likely reflects plasmid-mediated resistance associated with class 1 integron gene cassettes, with a possible contribution of plasmid-mediated quinolone resistance^40,41^. The second cluster, comprising amoxicillin/clavulanic acid, kanamycin, and cefotaxime, is consistent with β-lactamase production, most likely extended-spectrum β-lactamases of the CTX-M type^42^. The third cluster, consisting solely of chloramphenicol resistance, appears to represent an independent resistance determinant not linked to the main multidrug resistance clusters. However, these explanations remain tentative, as no molecular characterization of resistance genes was performed in the present work.

The material properties of playground equipment may play an important role in bacterial adhesion and persistence on surfaces^43–46^. Once on surfaces, pathogens can survive for hours to weeks, influenced by weather conditions and the type of material^26^. Non-porous plastics and stainless steel may support initial attachment, particularly under organic contamination^43^, while environmental conditions such as moisture, temperature, and UV exposure strongly modulate survival^26,44^. Wood and rubber-based surfaces can retain moisture and organic matter, which may facilitate longer bacterial persistence compared with smooth metal surfaces^44,46^. Overall, surface properties and environmental conditions jointly determine bacterial retention and potential transfer during contact^10,26,45,47^.

When assessing a child’s risk of bacterial infection from environmental sources, their age should also be taken into account, as it directly influences their behaviour on playgrounds and the types of equipment they prefer. For example, toddlers (children aged 1–3 years) prefer playing in sandboxes, on slides, spring riders, and interactive panels or educational toys. They require special attention because they may put sand, soil, or even faeces in their mouths during playground activities^48^. Older children (approximately 6–12 years) are more physically capable, independent, and seek challenges as well as social play. On playgrounds, they prefer activities that allow them to test their skills, compete, and collaborate with peers, often choosing climbing walls, ropes and nets, troll swings, rope bridges, balance beams, and pull-up bars. Statistical analysis of the data obtained herein indicated that sandboxes were the most important playground component likely to be contaminated with *E. coli*, followed by ground surfaces. The probability of detecting *E. coli* strains on climbing structures, swings, and spring riders or other attractions was comparable to the reference level. In contrast, the opposite trend was observed for *S. aureus*, although without a statistically significant association. Based on our results, it can be assumed that younger children are more likely to be exposed to *E. coli*, whereas older children are more likely to encounter *S. aureus*. However, no exposure assessment by age group was performed in this study; therefore, such interpretations remain speculative.

Here, we report a higher prevalence of *E. coli* compared to *S. aureus* in samples collected from playground areas. A similar pattern was reported in Greece, where a quantitative microbial risk assessment indicated a higher modelled probability of infection with *E. coli* than with *S. aureus* for a child spending one hour daily in a playground observation^8^. Furthermore, higher *E. coli* concentrations are typically observed in surface sand layers than in deeper layers^15^. This tendency of *E. coli* to survive better in surface sand highlights the importance of children practicing proper hand hygiene during and after play, not only in sandboxes but also following contact with surfaces contaminated with sand or soil. The importance of children’s hand hygiene is further emphasized by our findings for *S. aureus*. Most strains of *S. aureus* were isolated from plastic and metal surfaces, while only one was detected on a wooden surface. Moreover, we observed a tendency toward increased risk of *S. aureus* exposure when using swing structures, spring riders, or other attractions - particularly those made of plastic, which are frequently touched by hand. Due to the limited number of positive samples, these findings should be interpreted cautiously, as none of the examined variables demonstrated a statistically significant relationship with *S. aureus* presence. Nevertheless, the findings are consistent with observations reported for Hungarian playgrounds, where isolates were also predominantly found on synthetic materials, and *S. aureus* survival was highest on plastic and vinyl and lowest on wood^11^. Another study further demonstrated contamination of indoor surfaces such as crawl tubes, bars, and climbing walls^12^.

Resistance rates of *E. coli* isolates from swab samples were found to be higher than those obtained from soil samples^37^. Based on this observation, it was suggested that the probability of exposure to resistant bacterial strains is higher in areas frequently touched by children^37^. However, in the present study, this observation was not confirmed, as none of the modelled antimicrobial agents showed statistically significant associations between *E. coli* resistance and the tested variables (sampling location and substrate material).

Despite the relevance of these findings for environmental hygiene and playground safety, several limitations should be acknowledged. A key limitation of this study is that bacterial identification was based on MALDI-TOF MS, which enabled reliable species-level identification but did not allow characterization of *E. coli* pathotypes or detection of virulence genes. Therefore, the recovered *E. coli* isolates should be interpreted primarily as indicators of environmental contamination, and any implications regarding their specific pathogenic potential or public-health relevance remain speculative without additional molecular characterization. In addition, the study was conducted in a single geographical location (Lublin, Poland) and during one sampling period corresponding to the summer season. Consequently, seasonal variability in bacterial contamination and antimicrobial resistance patterns was not assessed, which may limit the generalizability of the findings. Moreover, although a total of 330 environmental samples were collected, *E. coli* was isolated from 50 samples, while *S. aureus* was detected in 15 samples. This relatively low number of positive isolates should be considered when interpreting the results, particularly for *S. aureus*. Taken together, these factors may affect the robustness of the analyses and the broader applicability of the observed prevalence and resistance patterns.

## Conclusion

The study aimed to assess the occurrence and distribution of *E. coli* and *S. aureus*, including antimicrobial-resistant isolates of potential pathogenic relevance, across different playground facilities, construction materials, and substrates, in order to characterize the microbiological quality of the playground environment. The results demonstrate that these bacteria, including resistant phenotypes, are present on selected playground surfaces and substrates, with variation observed across material types and sampling locations.

Assessment of surface contamination levels together with the physicochemical characteristics of playground materials may help to identify equipment most susceptible to pathogen persistence and inform future investigations of environmental persistence. However, this study did not directly assess survival dynamics or transmission pathways. Considering that the study was conducted in a single city (Lublin, Poland) and within a limited timeframe, there is a need for research with broader geographic coverage and extended duration.

Incorporating data on children’s behaviours - such as the frequency and duration of contact with specific playground equipment - could help future studies better estimate the potential transfer of bacteria of possible pathogenic relevance to the skin, mucous membranes, or minor abrasions during play. Future studies incorporating behavioural data, environmental factors, and molecular analyses would be required to better characterize transmission pathways and resistance mechanisms.

Longer bacterial persistence on surfaces and more frequent user contact may increase the likelihood of environmental exposure, although this study did not assess colonization or infection outcomes directly. Therefore, the present findings should be interpreted as descriptive and not indicative of health risk. While the results may inform future research directions, they do not allow for direct risk assessment or recommendations for intervention.

## Electronic Supplementary Material

Below is the link to the electronic supplementary material.

Supplementary Material 1

## Data Availability

The corresponding dataset is available in the OSF repository at [https://osf.io/8jsk4/] .

## References

[1] Salam, M. A. et al. Antimicrobial resistance: A growing serious threat for global public health. *Healthcare* **11**, 1946 (2023). 37444780 10.3390/healthcare11131946 PMC10340576

[2] Akram, F., Imtiaz, M., Haq, I. & ul Emergent crisis of antibiotic resistance: A silent pandemic threat to 21st century. *Microb. Pathog*. **174**, 105923 (2023). 36526035 10.1016/j.micpath.2022.105923

[3] Ballash, G. A., Parker, E. M., Mollenkopf, D. F. & Wittum, T. E. The One Health dissemination of antimicrobial resistance occurs in both natural and clinical environments. *J. Am. Vet. Med. Assoc.* **262**, 451–458 (2024). 38428137 10.2460/javma.24.01.0056

[4] Bengtsson-Palme, J. et al. Towards monitoring of antimicrobial resistance in the environment: For what reasons, how to implement it, and what are the data needs? *Environ. Int.* **178**, 108089 (2023). 37441817 10.1016/j.envint.2023.108089

[5] Ludden, C. et al. One Health genomic surveillance of Escherichia coli demonstrates distinct lineages and mobile genetic elements in isolates from humans versus livestock. *MBio* **10**, e02693–e02618 (2019). 30670621 10.1128/mBio.02693-18 PMC6343043

[6] Cave, R., Cole, J. & Mkrtchyan, H. V. Surveillance and prevalence of antimicrobial resistant bacteria from public settings within urban built environments: Challenges and opportunities for hygiene and infection control. *Environ Int* **157**, 106836 . 10.1016/j.envint.2021.106836 PMC8443212 34479136

[7] Łopucki, R. et al. Interspecies transmission of antimicrobial-resistant bacteria between wild birds and mammals in urban environment. *Vet. Microbiol.* **294**, 110130 (2024). 38820727 10.1016/j.vetmic.2024.110130

[8] Chatziprodromidou, I. P., Chatziantoniou, S., Vantarakis, G. & Vantarakis, A. Risk factor analysis of children’s exposure to microbial pathogens in playgrounds. *Risk Anal.* **42**, 334–343 (2022). 33969510 10.1111/risa.13752

[9] Hyytiäinen, H. K. et al. Crawling-induced floor dust resuspension affects the microbiota of the infant breathing zone. *Microbiome* **6**, 25 (2018). 29394954 10.1186/s40168-018-0405-8 PMC5797336

[10] Łopucki, R., Skowronek, M., Bilokinna, A., Martinez-de-Tejada, G. & Sadok, I. Awareness of microbiological safety in playgrounds amid rising antibiotic resistance. *Environ. Microbiol. Rep.* **17**, e70241 (2025). 41284386 10.1111/1758-2229.70241 PMC12643047

[11] Horváth, A. et al. High clonal diversity of Staphylococcus aureus isolates from children’s playgrounds in Hungary. *Sci. Rep.* **14**, 10021 (2024). 38693249 10.1038/s41598-024-60481-0 PMC11063029

[12] Thapaliya, D. et al. Prevalence and molecular characterization of Staphylococcus aureus and Methicillin-resistant S. aureus on children’s playgrounds. *Pediatr. Infect. Dis. J.* **38**, e43–e47 (2019). 29746375 10.1097/INF.0000000000002095

[13] Abdollahpour, N., Zendehbad, B., Alipour, A. & Khayatzadeh, J. Wild-birdfeces as a source of Campylobacterjejuniinfection in children’splaygrounds in Iran. *Food Control*. **50**, 378–381 (2015).

[14] Gurler, A. T. et al. Role of cat and dog faeces in the contamination of sandplaygrounds in public parks by Toxocara spp. *Med. Weter*. **76**, 441–445 (2020).

[15] Leri, A. C., Fassihi, G. E., Lundquist, M. J., Khan, M. & Arguin, M. L. Vertical stratification and seasonality of fecal indicator bacteria in New York City playground sandboxes. *Ecotoxicol. Environ. Saf.* **273**, 116152 (2024). 38417319 10.1016/j.ecoenv.2024.116152

[16] Abbas, G. et al. High rates of CTX-M group-1 extended-spectrum β-lactamasesproducing Escherichia coli from pets and theirowners in Faisalabad, Pakistan. *Infect. Drug Resist.* **12**, 571–578 (2019). 30881062 10.2147/IDR.S189884 PMC6411320

[17] Johnson, T. J. et al. Occurrence and potential transmission of extended-spectrum beta-lactamase-producing extraintestinal pathogenic and enteropathogenic Escherichia coli in domestic dog faeces from Minnesota. *Zoonoses Public. Health*. **69**, 888–895 (2022). 35799333 10.1111/zph.12985 PMC9796152

[18] Ortega-Paredes, D. et al. Multidrug-resistant Escherichia coli isolated from canine faeces in a public park in Quito, Ecuador. *J. Glob Antimicrob. Resist.* **18**, 263–268 (2019). 30980959 10.1016/j.jgar.2019.04.002

[19] Cuny, C., Layer-Nicolaou, F., Weber, R., Köck, R. & Witte, W. Colonization of dogs and their owners with Staphylococcus aureus and Staphylococcus pseudintermedius in households, veterinary practices, and healthcare facilities. *Microorganisms* **10**, 677 (2022). 35456729 10.3390/microorganisms10040677 PMC9024920

[20] Khairullah, A. R. et al. Pet animals as reservoirs for spreading methicillin-resistant Staphylococcus aureus to human health. *J. Adv. Vet. Anim. Res.* **10**, 1–13 (2023). 37155545 10.5455/javar.2023.j641 PMC10122942

[21] Loncaric, I. et al. Comparison of ESBL – and AmpC producing Enterobacteriaceae and methicillin-resistant Staphylococcus aureus (MRSA) isolated from migratory and resident population of rooks (Corvus frugilegus) in Austria. *PLoS One*. **8**, e84048 (2013). 24391878 10.1371/journal.pone.0084048 PMC3877145

[22] Sen, K. et al. American crows as carriers of extra intestinal pathogenic E. coli and avian pathogenic-like E. coli and their potential impact on a constructed wetland. *Microorganisms* **8**, 1595 (2020). 33081240 10.3390/microorganisms8101595 PMC7602749

[23] Cagnoli, G., Bertelloni, F., Ceccherelli, R. & Ebani, V. V. Antimicrobial resistance and pathotypes of Escherichia coli isolates from Yellow-Legged Seagulls (Larus michahellis) in central Italy. *Animals* **14**, 3048 (2024). 39518773 10.3390/ani14213048 PMC11545632

[24] Cantrell, M. E. et al. Hands are frequently contaminated with fecal bacteria and enteric pathogens globally: A systematic review and meta-analysis. *ACS Environ. Au*. **3**, 123–134 (2023).

[25] Martínez-Bastidas, T. et al. Detection of pathogenic micro‐organisms on children’s hands and toys during play. *J. Appl. Microbiol.* **116**, 1668–1675 (2014). 24524673 10.1111/jam.12473

[26] Wißmann, J. E. et al. Persistence of pathogens on inanimate surfaces: A narrative review. *Microorganisms* **9**, 343 (2021). 33572303 10.3390/microorganisms9020343 PMC7916105

[27] Sahin, C. K. & Onay, B. Alternative wood species for playgrounds wood from fruit trees. *Wood Res.* **65**, 149–160 (2020).

[28] Azzam, A. et al. Prevalence, trends, and molecular insights into colistin resistance among gram-negative bacteria in Egypt: a systematic review and meta-analysis. *Ann. Clin. Microbiol. Antimicrob.* **24**, 32 (2025). 40349047 10.1186/s12941-025-00799-3 PMC12065219

[29] Naidoo, N. Presence, pathogenicity, antibiotic resistance, and virulence factors of Escherichia coli: a review. *Bacteria* **4**, 16 (2025).

[30] Aloke, C. & Achilonu, I. Coping with the ESKAPE pathogens: Evolving strategies, challenges and future prospects. *Microb. Pathog*. **175**, 105963 (2023). 36584930 10.1016/j.micpath.2022.105963

[31] Miller, W. R. & Arias, C. A. ESKAPE pathogens: antimicrobial resistance, epidemiology, clinical impact and therapeutics. *Nat. Rev. Microbiol.* **22**, 598–616 (2024). 38831030 10.1038/s41579-024-01054-w PMC13147291

[32] Shariati, A. et al. Global prevalence and distribution of vancomycin resistant, vancomycin intermediate and heterogeneously vancomycin intermediate Staphylococcus aureus clinical isolates: a systematic review and meta-analysis. *Sci. Rep.* **10**, 12689 (2020). 32728110 10.1038/s41598-020-69058-z PMC7391782

[33] Lin, C. Y., Wang, J. H., Lin, K. H., Ho, Y. L. & Ho, C. M. Methicillin-resistant Staphylococcus aureus with reduced vancomycin susceptibility in Taiwan. *Tzu Chi Med. J.* **30**, 135–140 (2018). 30069120 10.4103/tcmj.tcmj_145_17 PMC6047320

[34] Sadok, I., Łopucki, R. & Skowronek, M. Protocol for the identification and antimicrobial susceptibility testing of bacteria isolated from playgrounds. *RepKUL* 1–9 (2024).

[35] Institute, C. and L. S. *Performance Standards for Antimicrobial Susceptibility Testing. CLSI supplement M100, 33rd ed.* (2023).

[36] Clinical and Laboratory Standards Institute. Performance Standards for Antimicrobial Susceptibility Testing. CLSI supplement M100. 34th ed. (2024).

[37] Caliskan, D., Bakkaloğlu, Z., Cevik, Y. N., Yildiz, S. S. & Kaskatepe, B. Maldi-TOF MS identification and antibiotic resistance of Escherichia coli isolated from playground. *Microb. Pathog*. **159**, 105155 (2021). 34418494 10.1016/j.micpath.2021.105155

[38] Mroczkowska, A. & Empel, J. Staphylococcus aureus-chorobotwórczość, antybiotykooporność, epidemiologia, perspektywa szczepionki. *Oporność na antybiotyki (Biuletyn informacyjny nr 1/2023). Zakład Epidemiologii i Mikrobiologii Klinicznej*. *Narodowy Instytut Leków* 1–9 (2023).

[39] Badura, A. et al. Prevalence, antibiotic resistance patterns and molecular characterization of Escherichia coli from Austrian sandpits. *Environ. Pollut*. **194**, 24–30 (2014). 25089889 10.1016/j.envpol.2014.07.007

[40] Carattoli, A. Plasmids and the spread of resistance. *Int. J. Med. Microbiol.* **303**, 298–304 (2013). 23499304 10.1016/j.ijmm.2013.02.001

[41] Partridge, S. R., Kwong, S. M., Firth, N. & Jensen, S. O. Mobile genetic elements associated with antimicrobial resistance. *Clin. Microbiol. Rev.* **31**, e00088–e00017 (2018). 30068738 10.1128/CMR.00088-17 PMC6148190

[42] Husna, A. et al. Extended-spectrum β-lactamases (ESBL): challenges and opportunities. *Biomedicines* **11**, 2937 (2023). 38001938 10.3390/biomedicines11112937 PMC10669213

[43] Merritt, K., Hitchins, V. M. & Brown, S. A. Safety and cleaning of medical materials and devices. *J. Biomed. Mater. Res.* **53**, 131–136 (2000). 10713558 10.1002/(sici)1097-4636(2000)53:2<131::aid-jbm1>3.0.co;2-i

[44] Tomičić, R., Tomičić, Z., Thaler, N., Humar, M. & Raspor, P. Factors influencing adhesion of bacteria Escherichia coli, Pseudomonas aeruginosa, Staphylococcus aureus and yeast Pichia membranifaciens to wooden surfaces. *Wood Sci. Technol.* **54**, 1663–1676 (2020).

[45] Wilks, S. A., Michels, H. & Keevil, C. W. The survival of Escherichia coli O157 on a range of metal surfaces. *Int. J. Food Microbiol.* **105**, 445–454 (2005). 16253366 10.1016/j.ijfoodmicro.2005.04.021

[46] Koutnik, V. S. et al. Children’s playgrounds contain more microplastics than other areas in urban parks. *Sci. Total Environ.* **854**, 158866 (2023). 36126714 10.1016/j.scitotenv.2022.158866

[47] Tarafdar, A., Oh, M. J., Nguyen-Phuong, Q. & Kwon, J. H. Profiling and potential cancer risk assessment on children exposed to PAHs in playground dust/soil: a comparative study on poured rubber surfaced and classical soil playgrounds in Seoul. *Environ. Geochem. Health*. **42**, 1691–1704 (2020). 31134396 10.1007/s10653-019-00334-2

[48] Simanjuntak, D. F. et al. A comparative pilot study on Gram-negative bacteria contaminating the hands of children living in urban and rural areas of Indonesia versus Germany – A suitable monitoring strategy for diarrhea risk assessment? *Front. Microbiol.* **14**, 1152411 (2023). 37077245 10.3389/fmicb.2023.1152411 PMC10106674

