# Supplementary material for: Patterns of occurrence and antimicrobial resistance of Escherichia coli and Staphylococcus aureus in public playgrounds
Source: Sci Rep. 2026 Apr 27;16:19416. doi: 10.1038/s41598-026-49426-x (PMC13287782; doi:10.1038/s41598-026-49426-x)
Supplement: Supplementary file 1 — Supplementary Material 1 [file 41598_2026_49426_MOESM1_ESM.docx]

**Patterns of occurrence and antimicrobial resistance of *Escherichia coli* and *Staphylococcus aureus* in public playgrounds**

Rafał Łopucki^1^,Marcin Skowronek^1^, Anna Bilokinna^2,3^, Aleksandra Niziołek^2^, Ilona Sadok^4,*^

^1^Department of Biomedicine and Environmental Research, Institute of Biological Sciences, Faculty of Medicine, Collegium Medicum, The John Paul II Catholic University of Lublin, Konstantynów 1J, 20-708 Lublin, Poland

^2^Biotechnology Scientific Club of the John Paul II Catholic University of Lublin, Faculty of Medicine, The John Paul II Catholic University of Lublin, Konstantynów 1I street, 20-708, Lublin, Poland

^3^Department of Microbiology, Institute of Soil Science and Plant Cultivation - State Research Institute, Czartoryskich 8, 24-100 Pulawy, Poland

^4^Department of Biomedical and Analytical Chemistry, Institute of Biological Sciences, Faculty of Medicine, Collegium Medicum, The John Paul II Catholic University of Lublin, Konstantynów 1J, 20-708 Lublin, Poland

*Corresponding author: ilona.sadok@kul.pl; phone: +48 81 445 46 18, Faculty of Medicine, Collegium Medicum, The John Paul II Catholic University of Lublin, Konstantynów 1J, 20-708 Lublin, Poland

Rafał Łopucki: lopucki@kul.pl, ORCID: 0000-0003-2137-8742

Marcin Skowronek: marskow@kul.pl, ORCID: 0000-0003-2069-0347

Anna Bilokinna: annabilokinna@gmail.com, ORCID: 0009-0003-6043-4054

Aleksandra Niziołek, olaniziolek2002@gmail.com, ORCID: 0009-0000-6886-0486

Ilona Sadok: ilona.sadok@kul.pl; ORCID: 0000-0003-1154-7581


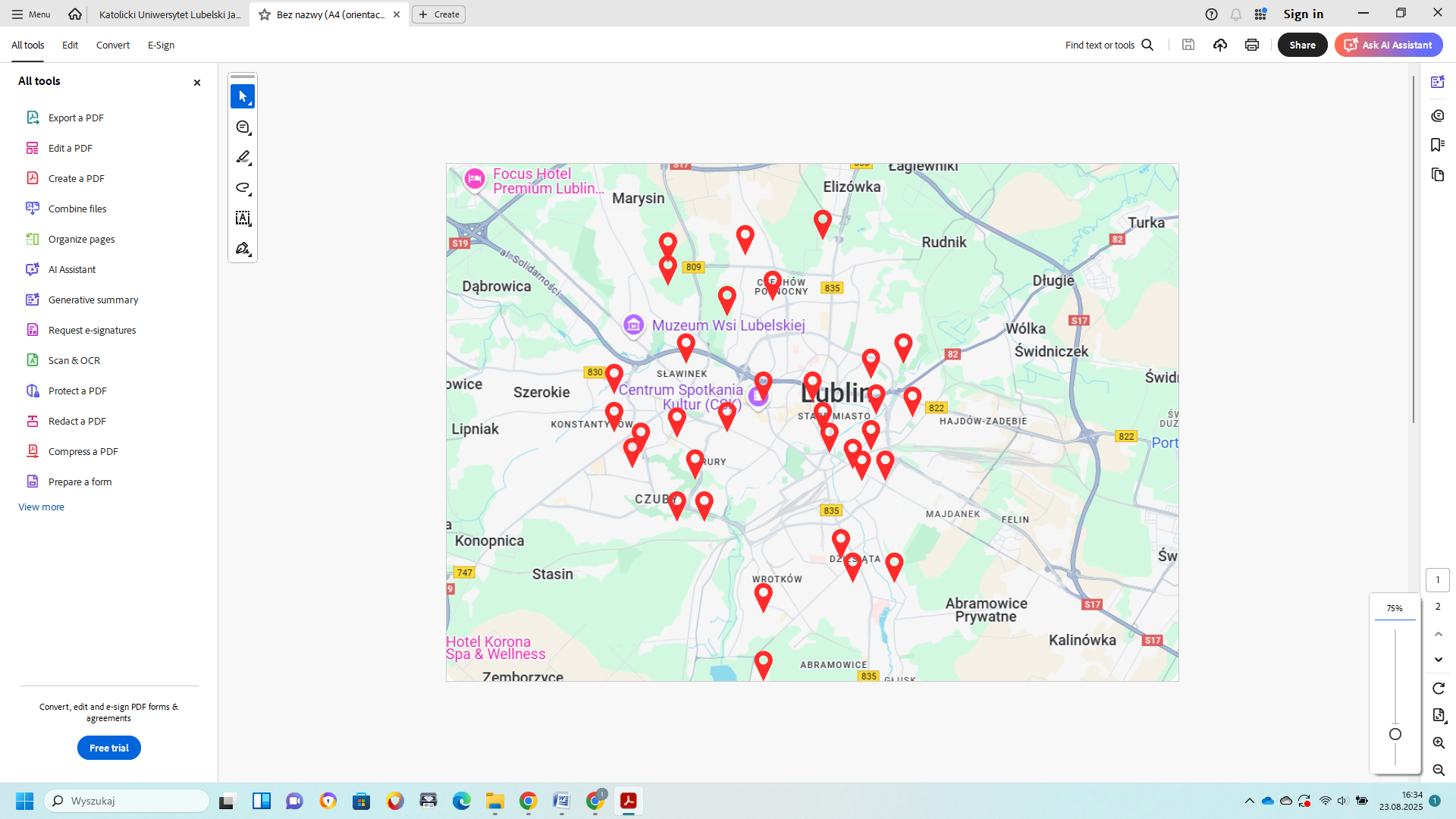


## Supplementary Figure S1. Localization of playgrounds across the city of Lublin, Poland.

**Supplementary Methods**

**Supplementary Methods S1. General Bayesian modelling framework**

To assess associations between playground characteristics and bacterial occurrence, antimicrobial resistance, and multidrug resistance, we used Bayesian generalized linear mixed-effects models (GLMMs) with a Bernoulli response distribution and logit link. Across all regression analyses, the fixed effects were sampling location and material type, both entered as categorical predictors using treatment coding. Reference levels were Bench for sampling location and Metal and mixed for material type. Bayesian estimation was selected because several outcomes were sparse, particularly for *S. aureus* occurrence and some antimicrobial resistance phenotypes, and this approach provided stable estimation together with directly interpretable uncertainty intervals.

Unless otherwise stated, results were summarized as posterior odds ratios (OR = exp[β]) with 95% credible intervals (CrI). Interactions between sampling location and material type were not evaluated because several cross-tabulated combinations were too sparse to support stable estimation.

**Supplementary Methods S2. Occurrence models for *E. coli* and *S. aureus***

To evaluate whether bacterial occurrence varied according to sampling location and material type, we fitted separate Bayesian mixed-effects logistic regression models for *E. coli* and *S. aureus*:

$$\mathrm{logit}\{P(Y_{ij}=1)\}=\beta_{0}+\beta_{1}{Sampling\_Location}_{ij}+\beta_{2}{Material\_Type}_{ij}+u_{j}$$

where $Y_{ij}=1$ if the target bacterium was detected in sample $i$from playground $j$, and $Y_{ij}=0$ otherwise. The random intercept for playground was specified as:

$$u_{j}\mathcal{\sim N}(0,\sigma_{\mathrm{playground}}^{2})$$

This structure accounted for clustering of samples within playgrounds. The occurrence analyses were based on 330 environmental samples collected from 33 playgrounds.

**Supplementary Methods S3. Antimicrobial resistance models**

Antimicrobial susceptibility results were first summarized descriptively as the numbers of isolates classified as susceptible (S), intermediate (I), or resistant (R) to each tested antimicrobial agent. Because only a small number of *S. aureus* isolates were recovered, inferential resistance models were constructed only for *E. coli*. For each antimicrobial agent, disk diffusion outcomes were dichotomized as R = 1 and S/I = 0. Only antimicrobial agents with at least 10 resistant isolates were included in model-based analyses. These were AMP10, S3-300, TE30, W5, S10, CIP5, and SXT25.

For each included antimicrobial agent, a separate Bayesian GLMM was fitted:

$$\mathrm{logit}\{P(Y_{ijk}=1)\}=\beta_{0}+\beta_{1}\mathrm{SamplingLocation}_{k}+\beta_{2}\mathrm{MaterialType}_{k}+u_{j}+v_{k}$$

where $Y_{ijk}=1$ if isolate $i$, obtained from sample $k$ in playground $j$, was resistant to the antimicrobial agent of interest, and $Y_{ijk}=0$ otherwise. The random effects were specified as:

$$u_{j}\mathcal{\sim N}(0,\sigma_{\mathrm{playground}}^{2}),v_{k}\mathcal{\sim N}(0,\sigma_{\mathrm{sample}}^{2})$$

A random intercept for playground ID accounted for clustering within playgrounds, whereas a random intercept for sample ID accounted for the non-independence of multiple *E. coli* isolates recovered from the same environmental sample when colonies with distinct morphology were observed on chromogenic medium.

**Supplementary Methods S4. Multidrug resistance (MDR) model**

The MDR analysis was restricted to *E. coli* isolates due to the limited number of recovered *S. aureus* strains. Antimicrobial agents were grouped into seven pharmacological classes: β-lactams (AMP10, AMC30, CTX30), carbapenems (IPM10, MEM10), aminoglycosides (CN10, K30, S10), tetracyclines (TE30), fluoroquinolones (CIP5), sulfonamides/trimethoprim (S3-300, W5, SXT25), and phenicols (C30). An isolate was classified as resistant to a drug class if it was resistant to at least one agent in that class. The variable $n\_class\_R$ was defined as the number of drug classes to which a given isolate was resistant. An isolate was classified as multidrug resistant (MDR) if it was resistant to three or more antimicrobial classes.

The probability of MDR was then analysed using the following Bayesian GLMM:

$$\mathrm{logit}\{P(\mathrm{MDR}_{ijk}=1)\}=\beta_{0}+\beta_{1}\mathrm{SamplingLocation}_{k}+\beta_{2}\mathrm{MaterialType}_{k}+u_{j}+v_{k}$$

where $u_{j}$ denotes the random intercept for playground ID and $v_{k}$ the random intercept for sample ID. This structure accounted both for clustering within playgrounds and for the non-independence of multiple isolates originating from the same environmental sample.

**Supplementary Methods S5. Resistance correlation and multivariate analyses**

To explore patterns of co-resistance among *E. coli* isolates, disk-diffusion results were converted into binary resistance indicators (R = 1, S/I = 0). Antimicrobial agents with ≤ 3 resistant isolates were excluded to reduce instability caused by extremely sparse categories. Pairwise phi correlations were calculated between resistance indicators, and a dissimilarity matrix was constructed as:

$$d_{ij}=1-\phi_{ij}$$

Hierarchical agglomerative clustering using complete linkage was applied to this dissimilarity matrix. The dendrogram was cut into three clusters, representing groups of antimicrobial agents with similar resistance profiles. These clusters were visualized in a correlation heatmap using the corrplot package [6].

In parallel, multiple correspondence analysis (MCA) was performed using the FactoMineR package [4] on the same binary resistance matrix. Coordinates of the resistant (“R”) categories were extracted for the first two dimensions and used to visualize relationships among resistance phenotypes in a low-dimensional space. MCA points were coloured according to hierarchical cluster membership, and labels were positioned to improve readability.

**Supplementary Methods S6. Priors, model fitting, diagnostics, and software**

For all Bayesian GLMMs, weakly informative priors were used: regression coefficients: $\beta\sim N(0,1)$; intercept: Student’s $t(3,0,2)$; and standard deviations of random effects: Cauchy (0,1). Each model was fitted using four Markov chains, with 4000 iterations per chain, including 2000 warm-up iterations and 2000 post-warm-up sampling iterations, yielding 8000 posterior samples per model. Convergence was assessed using standard MCMC diagnostics, including visual inspection of trace plots, $\hat{R}<1.01$, and adequate effective sample sizes. For the occurrence models, effective sample sizes for fixed effects exceeded 1000; for the MDR model, all parameters showed $\hat{R}\leq1.01$and bulk effective sample sizes > 400.

All analyses were performed in R(v.4.5.0) [1]. Bayesian models were fitted using the brms package (v.2.22.0)[2], interfaced with Stan(v.2.32.7) [3]. MCA was performed using FactoMineR [4], and figures were prepared using ggplot2 (v.3.5.2) [5]. Correlation heatmaps were generated using corrplot [6]. Forest plots displayed posterior OR estimates with 95% CrI on a logarithmic scale.

**References**

[1]. R Core Team. *R: A Language and Environment for Statistical Computing*. R Foundation for Statistical Computing, Vienna, Austria,https://www.r-project.org/ (2025).

[2]. Bürkner, P.C. brms: An R package for Bayesian multilevel models using Stan. *J. Stat. Softw.* 80(1), 1–28 (2017).

[3]. Carpenter, B., Gelman, A., Hoffman, M. D., Lee, D., Goodrich, B., Betancourt, M., … Riddell, A. Stan: A probabilistic programming language. *J. Stat. Softw.* 76(1), 1–32 (2017).

[4]. Lê, S., Josse, J. & Husson, F. FactoMineR: A package for multivariate analysis. *J. Stat. Softw.* 25(1), 1–18 (2008).

[5]. Wickham, H. *ggplot2: Elegant Graphics for Data Analysis*. Springer-Verlag, New York (2016).

[6]. Wei, T. & Simko, V. *R package ‘corrplot’: Visualization of a Correlation Matrix* (Version 0.95, 2024).

## Supplementary Figure S2. Posterior odds ratios (ORs) for resistance to ampicillin (AMP10) among *E. coli* isolates by sampling location, estimated using a Bayesian GLMM.

Points represent posterior means; horizontal lines denote 95 % credible intervals (CrI). All intervals cross the null effect (OR = 1), indicating no statistically credible differences between locations.

## Supplementary Figure S3. Posterior odds ratios (ORs) for resistance to CIP5 among *E. coli* isolates by substrate material, estimated using a Bayesian GLMM.

Points show posterior means; horizontal bars indicate 95 % credible intervals. No material type was credibly associated with resistance. Soil and rubber surfaces had the highest point estimates but with wide and inconclusive CrIs.

## Supplementary Figure S4. Posterior odds ratios (ORs) for multidrug resistance (MDR) in *E. coli* isolates, estimated using a Bayesian generalized linear mixed model (GLMM).

Fixed effects include sampling location and substrate material; reference levels are bench (location) and metal/mixed surfaces (material). Points represent posterior means and horizontal lines indicate 95 % credible intervals (CrI). All intervals overlap the null value (OR = 1), indicating no statistically credible associations between MDR and environmental factors.

**Supplementary Figure S5.** Multiple Correspondence Analysis (MCA) biplot showing the spatial distribution of antimicrobial resistance phenotypes. Each point represents an individual antimicrobial agent, positioned based on shared resistance profiles across isolates. Cluster membership (k = 3) was assigned via hierarchical clustering on the φ-correlation matrix and is indicated by color. The analysis revealed: (1) a multidrug-resistant (MDR) cluster (red), including AMP10, TE30, S3-300, SXT25, W5, S10, and CIP5; (2) a β-lactam-associated cluster (blue), comprising AMC30, K30, CTX30, despite notable dispersion on the MCA plane; and (3) an isolated resistance phenotype (green), represented solely by C30. Axes represent the first two dimensions of the MCA, explaining 58.8% of total variance.
